# The association between genetic variants in lactotransferrin and dental caries: a meta- and gene-based analysis

**DOI:** 10.1186/s12881-020-01029-7

**Published:** 2020-05-27

**Authors:** Xueyan Li, Yi Su, Di Liu, Jingyun Yang

**Affiliations:** 1 Department of Stomatology, Eye & Ent Hospital of Fudan University, Shanghai, China; 2 Beijing Key Laboratory of Clinical Epidemiology, School of Public Health, Capital Medical University, Beijing, China; 3 Division of Statistics, School of Economics, Shanghai University, 99 Shangda Rd, Baoshan Dist, Shanghai, 200444 China; 4 Research Center of Financial Information, Shanghai University, Shanghai, China; 5 Rush Alzheimer’s Disease Center, Rush University Medical Center, Chicago, IL USA; 6 Department of Neurological Sciences, Rush University Medical Center, Chicago, IL USA

**Keywords:** *LTF*, Dental caries, Polymorphism, Meta-analysis

## Abstract

**Background:**

The pathogenesis of dental caries remains unclear, with increasing evidence suggesting that genetic susceptibility plays an essential role. Previous studies have reported the association between genetic polymorphisms in lactotransferrin (*LTF*) and the risk of dental caries with inconsistent results.

**Methods:**

A systematic literature search of the PubMed, Cochrane Library, HuGE and Google Scholar databases was performed by two authors independently for papers published before December 5, 2019 on the association between genetic variants in *LTF* and the risk of dental caries. We adopted the subsequent inclusion criteria to assess study eligibility: 1) The studies were based on human subjects; 2) the presence of dental caries should be screened for in both the case group and the control group; and 3) genotype data on variants in *LTF* were available in both the case group and the control group. We calculated odds ratios (ORs) and the corresponding 95% confidence intervals (CIs) by using random-effects models to assess the association of genetic variants in *LTF* with the risk of dental caries. We also performed a gene-based analysis to explore the joint association of multiple genetic variants in *LTF* with the risk of dental caries.

**Results:**

Our systematic literature search identified six relevant papers for analysis. We found no significant association between rs1126478 and the risk of dental caries when meta-analysing the genotype distribution between subjects with dental caries and those without dental caries (additive model: OR = 1.41; 95% CI = 0.98–2.02; *P* = 0.065). However, further analysis indicated that rs1126478 was associated with dental risk in subjects who had moderate or severe dental caries compared to those without dental caries (*P* < 0.0001). The gene-based analysis indicated that multiple genetic variants in *LTF* were jointly associated with the risk of dental caries (*P* = 0.002).

**Conclusions:**

The present meta-analysis revealed some evidence of the association between rs1126478 and dental caries and that multiple genetic variants in *LTF* are jointly associated with the risk of dental caries. Our findings need to be validated by larger studies that adjust for important confounding factors for the risk of dental caries.

## Background

Dental caries, also known as caries or dental decay, refers to the localised destruction of dental hard tissues [1, 2]. Dental caries not only affects the appearance and function of teeth but can have downstream effects on health. Although epidemiological studies have indicated that the lifetime prevalence of dental caries has decreased over the past four decades, the decrease mainly occurred in high-income countries (HICs) [3]. Dental caries remains very common despite the adoption of various preventive measures. For example, the National Center for Health Statistics has estimated that the prevalence of total dental caries, including untreated and treated cases, was 45.8% in primary or permanent teeth among youth aged 2–19 for 2015–2016 in the United States [4].

Dental caries is a complex multi-factorial disease resulting from long-term interaction between acid-producing bacteria and multiple biological, physical and environmental risk factors, such as salivary flow, diet, oral hygiene and fluoride exposure [5, 6]. However, these variables alone cannot entirely explain the onset and development of dental caries. Increasing evidence suggests that genetic susceptibility plays an essential role in the etiological mechanisms of dental caries [7].

Saliva forms the most important external environment for dental health. It contains a variety of antibacterial proteins that can effectively inhibit the accumulation and adhesion of oral bacteria, thereby preventing the incidence of dental caries [8]. Lactotransferrin (*LTF*) is an important iron-binding glycoprotein produced by saliva, and it can affect the occurrence and development of dental caries in a variety of ways [9].

The *LTF* gene is located on chromosome 3 at position 3p21 and is organised into 17 exons, with a size between 23 and 35 kb [10]. Multiple studies have examined the association between genetic variants in *LTF* and dental caries. A polymorphism (140A/G, rs1126478), located in the second exon, is responsible for the substitution of a lysine (Lys) with an arginine (Arg) at position 29 (or 47 depending on nomenclature) in the antimicrobial region. This single nucleotide polymorphism (SNP) might influence the expression level and function of the *LTF* protein [11]. The association between the rsl126478 polymorphism and the risk of dental caries has been examined in several previous studies, with inconsistent findings [12–15]. Meanwhile, the association of other genetic variants in *LTF* has been reported in connection with the risk of dental caries [15, 16]. Therefore, we performed this meta-analysis to examine the association between rsl126478 and dental caries. We also performed a gene-based analysis to explore the joint association of multiple genetic variants in *LTF* with the risk of dental caries.

## Methods

Due to the systematic review and meta-analytic nature of this study, ethical approval and informed consent statements are not required.

### Eligibility criteria

We adopted the subsequent inclusion criteria to assess study eligibility: 1) The studies were based on human subjects; 2) the presence of dental caries should be screened for in both the case group and the control group; and 3) genotype data on variants in *LTF* were available in both the case group and the control group. When there were multiple studies using overlapping data, we chose the one that had a larger sample size.

### Search strategy

A systematic literature search of the PubMed, Cochrane Library, HuGE and Google Scholar databases was performed by two authors (XL and JY) independently for papers published before December 5, 2019. We used a combination of keywords as appropriate, including LFT, lactotransferrin, dental caries, tooth decay and cavities.

All potentially relevant publications were retrieved to assess study eligibility. The references in all identified studies were also checked for studies that might have been missed during the initial literature search. Google Scholar’s ‘cited by’ tool was also used to search for potential eligible publications that cited the studies identified in the literature search. Literature search was performed independently by the two authors with a limitation to studies published in English. Any disagreement regarding study eligibility was resolved by group discussion (XL, YS and JY).

### Data extraction

Following a pre-specified protocol, the following data were extracted independently by two authors (DL and JY): name of the first author, year of publication, basic characteristics of the study participants, including sample size, mean age, distribution of gender, race/country of origin of the participants, screening method for dental caries, and genotype data for patients with and without dental caries or for patients with different severities of dental caries. Any discrepancies that emerged in the data extraction were resolved in group meetings. Newcastle–Ottawa Scale (NOS) was used to assess the quality of the included studies, which was done by two authors (DL and JY) independently [17].

### Data analysis

Hardy–Weinberg equilibrium (HWE) was satisfied in the control group of all the included studies. We calculated odds ratios (ORs) and the corresponding 95% confidence intervals (CIs) by using random-effects models to assess the association between genetic variants in *LTF* with the risk of dental caries. We performed analysis by assuming different genetic models, including additive, allelic, dominant, recessive and co-dominant genetic models. We used I^2^ to assess between-study heterogeneity, and a funnel plot and Egger’s test to assess publication bias.

### Gene-based analysis

Since it is likely that multiple genetic variants in *LTF* jointly contribute to the risk of dental caries, we performed a gene-based analysis by combining *P*-values for association of individual genetic variants in *LTF* to assess the joint association. This was done by using four *P*-value combination methods, including the Fisher’s method [18], the Simes method [19], the modified inverse normal method [20] and the modified truncated product method (TPM) [21, 22]. For the modified TPM, we calculated a weighted version and a unweighted version, where the former ignores the difference in sample sizes while the latter uses the sample size for each study as the weight [21]. Detailed information of the four *P*-value combination methods were given elsewhere [22]. We ran 100,000 simulations to estimate the correlation of the *P*-values in the calculation of the *P*-value for the modified TPM.

### Sensitivity/additional analysis

We performed sensitivity analysis by excluding low-quality studies (NOS < six stars); we also examined the association stratified by severity of dental caries using data from each individual study.

All statistical analyses were performed using R (https://www.r-project.org/) and MATLAB (The MathWorks, Inc., Natick, MA, USA). A *P*-value < 0.05 was considered statistically significant. This study was reported according to the PRISMA guidelines [23].

## Results

### Study selection and characteristics

**Fig. 1:**
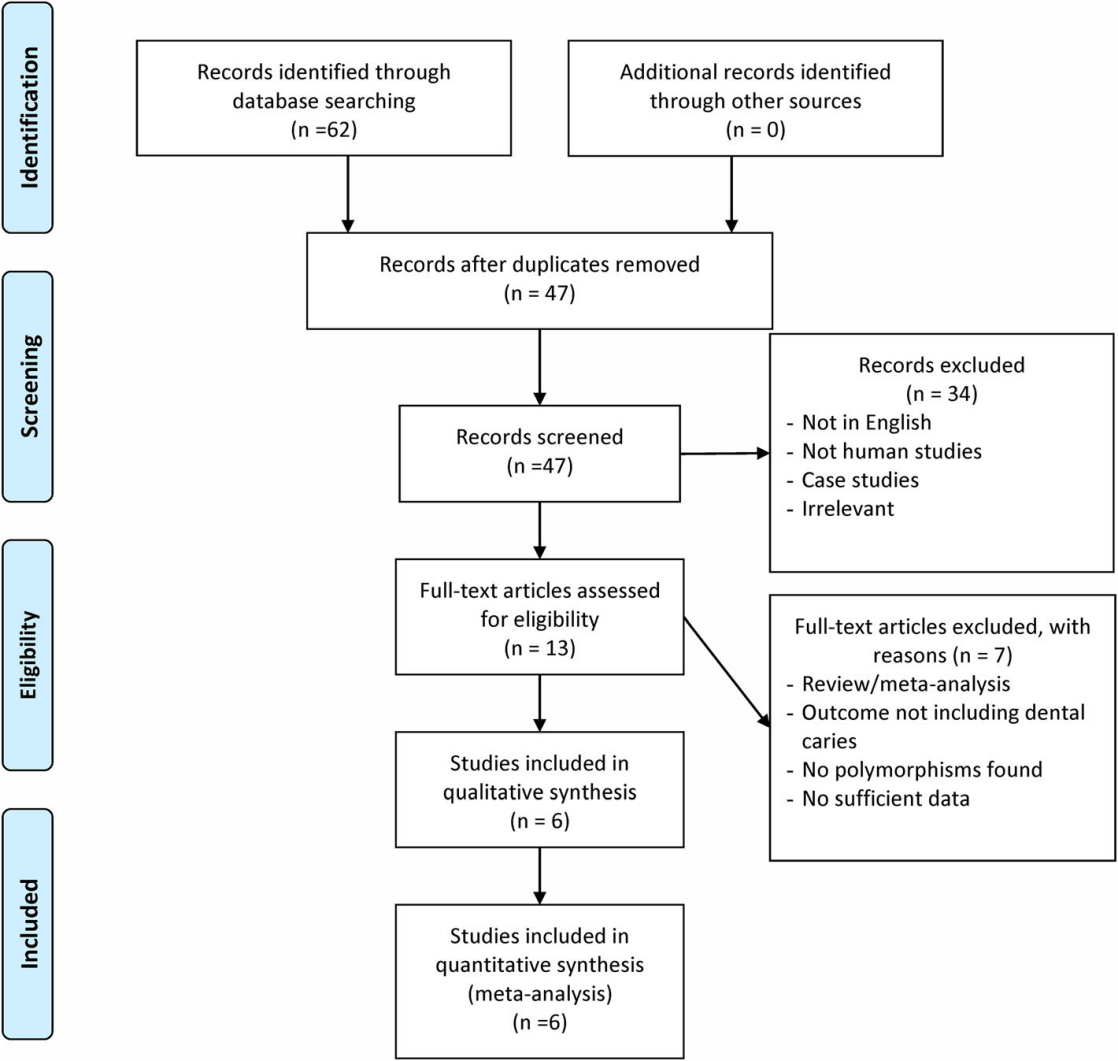
Flow diagram of the selection process of the studies included in the meta-analyses. Note: Please see the Methods section for additional details

The process of selection of eligible studies is shown in Fig. 1. We identified 47 potential publications through our initial search. After screening the abstracts, we excluded 34 publications because they were not in English, were not about human subjects, were case studies or were irrelevant. This left 13 studies that were retrieved for more detailed evaluation. We excluded an additional five studies because they were reviews or meta-analyses or because the outcomes did not include dental caries. This resulted in eight potentially relevant publications. We then excluded two more studies because there was insufficient data or no polymorphisms were detected, resulting in six publications that were finally included in our analyses [12–16, 24]. It should be noted that two studies used overlapping data [16, 24], with one study covering several new genetic variants in *LTF* other than rs1126478. Therefore, in the main meta-analysis for the association of rs1126748 with dental caries, we used the study with a larger sample size [24], while the analysis for other variants used data from the other study [16]. In summary, the main meta-analyses of rs1126748 included four studies [12–14, 24] with 1066 subjects having dental caries and 736 subjects having no caries. Data for other genetic variants, including rs1126477, rs6441989, rs2073495 and rs11716497, came from individual studies [15, 16].

All included publications were published after 2010. Table 1 shows the basic characteristics of the six included studies. Most of the studies are of good quality, except one the sample size of which is very limited [13].

**Table 1:** Basic characteristics of all the studies included in the analyses

Study	Year of publication	Country/origin	Ethnicity	Dental caries			Control			Diagnosis of dental caries	NOS
				n	Age	Male (%)	n	Age	Male (%)		
Azevedo et al.	2010	Brazil	Caucasian	62	12	–	48	12	–	DMFT	7
Fine et al.	2013	US	Mixed	17	–	–	33	–	–	Radiolucency	5
Volckova et al.	2014	European countries	Caucasian	482	11–13	50.00	155	11–13	52.90	DMFT	7
Doetzer et al.	2015	Brazil	Mixed	346	12	45.3	331	12	44.1	DMFT	9
Wang et al.	2017	China	Asian	505	3.48 ± 0.58	52.70	500	3.64 ± 0.33	49.00	DMFT	8
Wang et al.	2018	China	Asian	507	3.52 ± 0.51	52.64	403	3.57 ± 0.33	48.90	DMFT	8

Data for age were presented as mean, mean ± SD or range

*SD* Standard deviation, *NOS* the Newcastle–Ottawa scale, *DMFT* Decayed, missing and filled teeth index

### Assessment of publication Bias

**Fig. 2:**
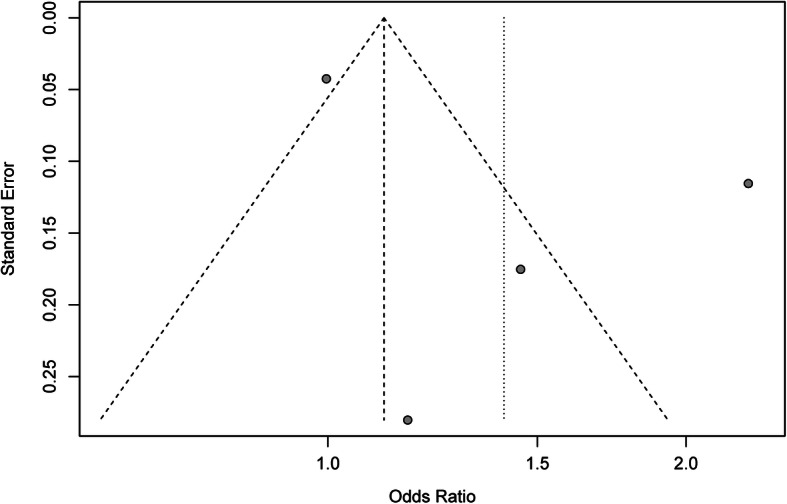
Funnel plot for meta-analysis of the association of rs1126478 with dental caries assuming an additive model. The x-axis is the odds ratio, and the y-axis is the standard error of estimated effect of rs1126478 on risk of dental caries. The vertical line in the figure represents the overall estimated odds ratio. The two diagonal lines represent the pseudo 95% confidence limits of the effect estimate

For simplicity, we mainly reported results assuming an additive model for the meta-analysis of rs1126478. Analysis results obtained assuming other genetic models can be found in Table 2. There was no evidence of publication bias for the meta-analysis of the four included studies (*P* = 0.374; Fig. 2) or for the meta-analyses assuming other genetic models (all *P-*values > 0.05). There was no evidence of publication bias in the sensitivity analysis which excluded the study using adult data [13] (*P* = 0.402).

**Table 2:** Association of rs1126748 with dental caries under different genetic models

Genetic models	OR (95% CI)	***P***
**Additive (reference, A)**	1.41 (0.98–2.02)	0.065
**Allelic (G vs. A)**	1.41 (0.48–4.13)	0.386
**Dominant (GG + GA vs. AA)**	1.32 (0.43–4.03)	0.484
**Recessive (GG vs. GA + AA)**	1.55 (0.44–5.38)	0.348
**Co-dominant (GA vs. GG + AA)**	0.97 (0.72–1.32)	0.778

*OR* Odds ratio, *CI* Confidence interval

### Association of rs1126748 with dental caries

**Fig. 3:**
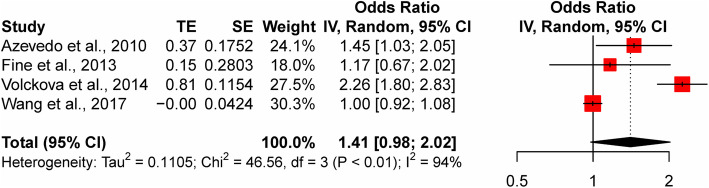
Forest plot for meta-analysis of the association of rs1126478 with dental caries. Each study is represented by a square whose area is proportional to the weight of the study. The overall effect from meta-analysis is represented by a diamond whose width represents the 95% CI for the estimated OR. OR, odds ratio; CI, confidence interval

Assuming an additive genetic model, we found no association of rs1126748 in *LTF* with the risk of dental caries in a random-effects meta-analysis including the four studies (OR = 1.41, 95% CI: 0.98–2.02, *P* = 0.065; Fig. 3). There existed high heterogeneity among the included studies (I^2^ = 93.6%, *P* < 0.0001). We found no association in the meta-analysis excluding the study that used adult data [13] (OR = 1.47, 95% CI: 0.92–2.35, *P* = 0.106; I^2^ = 95.7%, *P* for heterogeneity = < 0.0001; online supplementary Figure 1). A meta-analysis assuming other genetic models also revealed no significant association of rs1126748 with the risk of dental caries (allelic G vs. A: OR = 1.41, 95% CI: 0.48–4.13, *P* = 0.386; dominant GG + GA vs. AA: OR = 1.32, 95% CI: 0.43–4.03, *P* = 0.484; recessive GG vs. GA + AA: OR = 1.55, 95% CI: 0.44–5.38, *P* = 0.348; and co-dominant GA vs. AA+GG: OR = 0.97, 95% CI: 0.72–1.32, *P* = 0.778; online Supplementary Figure 2).

Two studies provided genotype data for patients with different severities of dental caries, and we examined the association stratified by severity of dental caries using data from each individual study. One study [14] categorised dental caries into low (decayed, missing and filled teeth index [DMFT] = 1), moderate (2 ≤ DMFT ≤3) and high (DMFT ≥4) groups. Compared with the control group, we found a significant association in patients with a low level of dental caries (OR = 0.65, 95% CI: 0.47–0.90, *P* = 0.008) but no significant association in patients with a moderate level of dental caries (OR = 1.04, 95% CI: 0.80–1.34, *P* = 0.792) or in patients with a severe level of dental caries (OR = 1.20, 95% CI: 0.94–1.54, *P* = 0.148). However, the association was significant when combining patients with a moderate level of dental caries and patients with a severe level of dental caries (OR = 1.88, 95% CI: 1.50–2.37, *P* < 0.0001). Another study [16] provided genotype data for patients with a relatively severe level of dental caries and categorised patients into moderate (8 ≤ DMFF ≤12) and severe (13 ≤ DMFT ≤20) groups. Compared with the control group, we found a significant association in both patients with a moderate level of dental caries (OR = 0.72, 95% CI: 0.65–0.80, *P* < 0.0001) and patients with a severe level of dental caries (OR = 0.78, 95% CI: 0.70–0.87, *P* < 0.0001).

### Association of other genetic variants in LTF with dental caries

A few studies provided genotype data for other genetic variants in *LTF*, and we examined their association with dental caries. The results are summarised in Table 3. Specifically, there was a significant association of the risk of dental caries with rs1126477 (OR = 1.17, 95% CI: 1.06–1.29, *P* = 0.002) but no significant association with rs6441989 (*P* = 0.697), rs2073495 (*P* = 0.941) and rs11716497 (*P* = 0.575).

**Table 3:** Association of other genetic variants in *LTF* with dental caries

Genetic variants	Study	OR (95% CI)	P
**rs11216477**	Wang et al., 2018 [16]	1.17 (1.06–1.29)	0.002
**rs6441989**	Doetzer et al., 2015 [15]	1.02 (0.92–1.14)	0.697
**rs2073495**	Doetzer et al., 2015 [15]	0.99 (0.86–1.15)	0.941
**rs11716497**	Doetzer et al., 2015 [15]	1.04 (0.90–1.21)	0.575

*LTF* Lactoferrin, *OR* Odds ratio, *CI* Confidence interval

### Gene-based analysis

To assess the joint association of multiple genetic variants in *LTF* with dental caries, we used results from our meta-analysis and results for other genetic variants from single studies. All the four *P*-value combination methods indicated a significant joint association of genetic variants in *LTF* with the risk of dental caries (Table 4). Specifically, the unweighted TPM that took into account the correlation among the *P*-values indicated a joint association of 0.001, and the weighted TPM that further took into account the sample sizes of each study indicated a joint association of 0.0007.

**Table 4:** Gene-based analysis of association of genetic variants in *LTF* with the risk of dental caries

Gene	Fisher	Simes	Inverse	TPM (unweighted)	TPM (weighted)
*LFT*	0.028	0.009	0.069	0.001	0.0007

*LTF* Lactotransferrin, *TPM* The modified truncated product method

## Discussion

In this paper, we examined the association of multiple genetic variants in *LTF* with the risk of dental caries using meta- and gene-based analyses. We found no significant association of rs1126478 with the risk of dental caries when comparing subjects with dental caries with those without dental caries. However, further analysis indicated that rs1126478 was associated with dental risk in subjects who had moderate or severe dental caries compared to those without dental caries. The gene-based analysis indicated that multiple genetic variants in *LTF* showed joint association with the risk of dental caries.

*LTF* can block the formation of biofilm by stimulating twitching, which results in bacteria wandering and prevents the formation of bacteria clusters and biofilms [25]. In addition to the antibacterial function, *LTF* is also involved in various physiological functions, such as iron absorption and modulated immune responses [9], thereby affecting the development of dental caries. The G allele of the SNP rs1126478 results from the substitution of lysine with arginine at position 29. The arginine variation of *LTF* has a weaker antibacterial effect on Gram-positive bacteria. A previous in vitro study indicated that LTF from whole saliva derived from recombinant human LTF with the lysine (K) variant of rs1126478 in *LTF* killed mutants streptococci associated with caries by more than 1 log compared to the arginine (R) variant [11]. However, the exact role of this genetic variant in the association with the risk of dental caries remains undetermined.

Of the four studies included in the meta-analysis of rs1126478, three [12–14, 24] indicated increased susceptibility of the G allele in association with the risk of dental caries, although the effect of the G allele was not significant in one study that has a very limited sample size and a wide CI of the estimated effect [13]. In contrast, the other study seems to indicate a smaller or even a reverse effect of the G allele (OR = 0.997 in the additive model) [24]. There were differences among the studies, such as age distribution and race/ethnicities of the study participants. It is unclear whether the variation of the effect of G allele does exist or whether it is affected by other confounding factors, which warrant further study.

We also explored the association between dental caries risk and other genetic variants in *LTF*, including rs1126477, rs6441989, rs2073495 and rs11716497. These genetic variants were less well studied compared to the other SNP, rs1126478. Our analysis indicated a significant joint association of multiple genetic variants in *LTF* with the risk of dental caries. However, caution should be exercised in interpreting these results because the effect of each genetic variant was estimated using data from single studies [15, 16], and therefore the estimates might be subject to bias. Another study searched for genetic variations in the promoter region of *LTF* but failed to identify any polymorphisms [26]. Future studies that target more genetic variants in *LTF* are greatly needed to examine whether the risk of dental caries is also affected by other genetic variants in *LTF*.

Our study has some limitations. First, the number of studies for the meta-analysis of rs1126478 is limited, despite the systematic literature search. Our findings need to be validated by future studies that have larger sample sizes. Second, we observed significant heterogeneity among the included studies. The participants in the included studies were of different ethnic backgrounds. This might partly explain the different genetic structures of the included participants. For example, three of the four studies in the meta-analysis showed G was the minor allele of rs1126478 in the control group having a minor allele frequency ranging from 0.26–0.39 [12–14], while a study of Chinese children [24] indicated that G was the major allele in the control group having a frequency of 0.66. The sample sizes in some studies were very limited [12, 13]. All these factors could contribute to the observed heterogeneity of the studies. However, due to the limited availability of data from each individual study, we could not track the exact source of the heterogeneity. Meta-regression is also not feasible and/or meaningful, again due to the limited number of studies [27] and the availability of relevant data from the included studies. Finally, we could not control for important factors that may affect the risk of dental caries due to a lack of data for individual subjects. The estimated effect of genetic variants in *LTF* on dental caries risk might be biased due to confounding, thereby influencing the validity of any meta-analysis that used unadjusted results. Therefore, such confounding factors should be taken into account in future studies to more accurately disentangle the exact relationship between *LTF* and the risk of dental caries.

## Conclusions

In summary, the present meta-analysis revealed no significant association of the genetic variant rsl126478 in *LTF* with the risk of dental caries. However, the relationship might vary depending on the severities of dental risk. Moreover, multiple genetic variants in *LTF* showed a joint association with the risk of dental caries. Our findings need to be validated by larger studies that take into account important confounding factors for the risk of dental caries. Prospective studies that adjust for other important relevant factors, such as diet and microbial and host characteristics, are also useful to elucidate the relationship between genetic variants in *LTF* and dental caries.

## Supplementary information

**Additional file 1: Figure S1.** Forest plot for meta-analysis of the association of rs1126478 with dental caries with adult data excluded. Each study is represented by a square whose area is proportional to the weight of the study. The overall effect from meta-analysis is represented by a diamond whose width represents the 95% CI for the estimated OR. OR, odds ratio; CI, confidence interval.

**Additional file 2: Figure S2.** Forest plot for meta-analysis of the association of rs1126478 with dental caries using other genetic models. A) Allelic model; B) Dominant model; C) Recessive model and D) Co-dominant model. Each study is represented by a square whose area is proportional to the weight of the study. The overall effect from meta-analysis is represented by a diamond whose width represents the 95% CI for the estimated OR. OR, odds ratio; CI, confidence interval.

## Data Availability

All data generated or analysed during this study are included in this published article and its supplementary information files.

## Abbreviations

LTF: Lactotransferrin
HIC: High income countries
CI: Confidence interval
OR: Odds ratio
NOS: Newcastle–Ottawa scale
HWE: Hardy–Weinberg equilibrium
DMFT: Decayed, missing and filled teeth index
TPM: Truncated product method
