# Supplementary figures and images for: The association between genetic variants in lactotransferrin and dental caries: a meta- and gene-based analysis
Source: BMC Med Genet. 2020 May 27;21:114. doi: 10.1186/s12881-020-01029-7 (PMC7251739; doi:10.1186/s12881-020-01029-7)

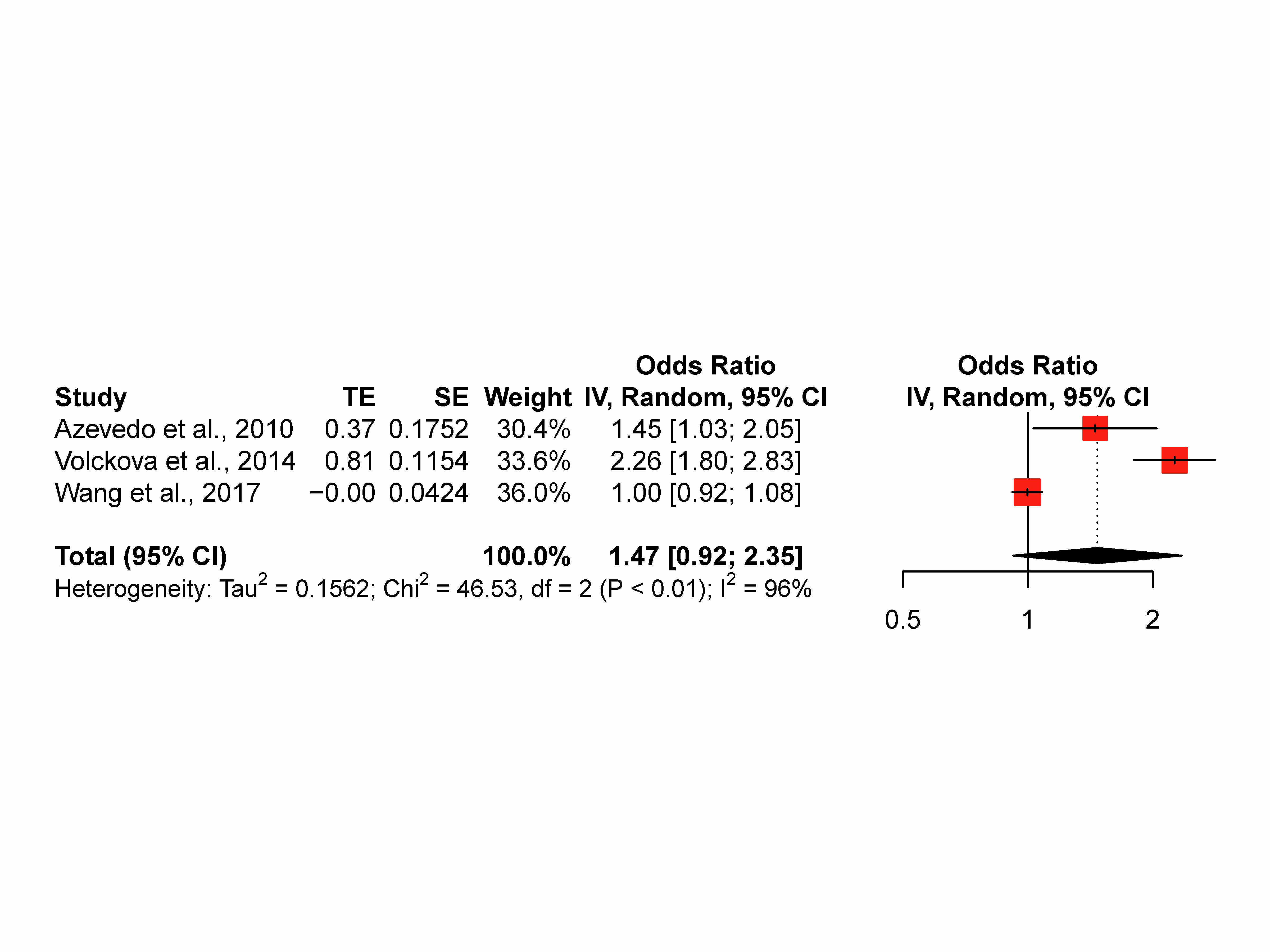

Supplement: Supplementary file 1 — Additional file 1: Figure S1. Forest plot for meta-analysis of the association of rs1126478 with dental caries with adult data excluded. Each study is represented by a square whose area is proportional to the weight of the study. The overall effect from meta-analysis is represented by a diamond whose width represents the 95% CI for the estimated OR. OR, odds ratio; CI, confidence interval. [file 12881_2020_1029_MOESM1_ESM.tif]

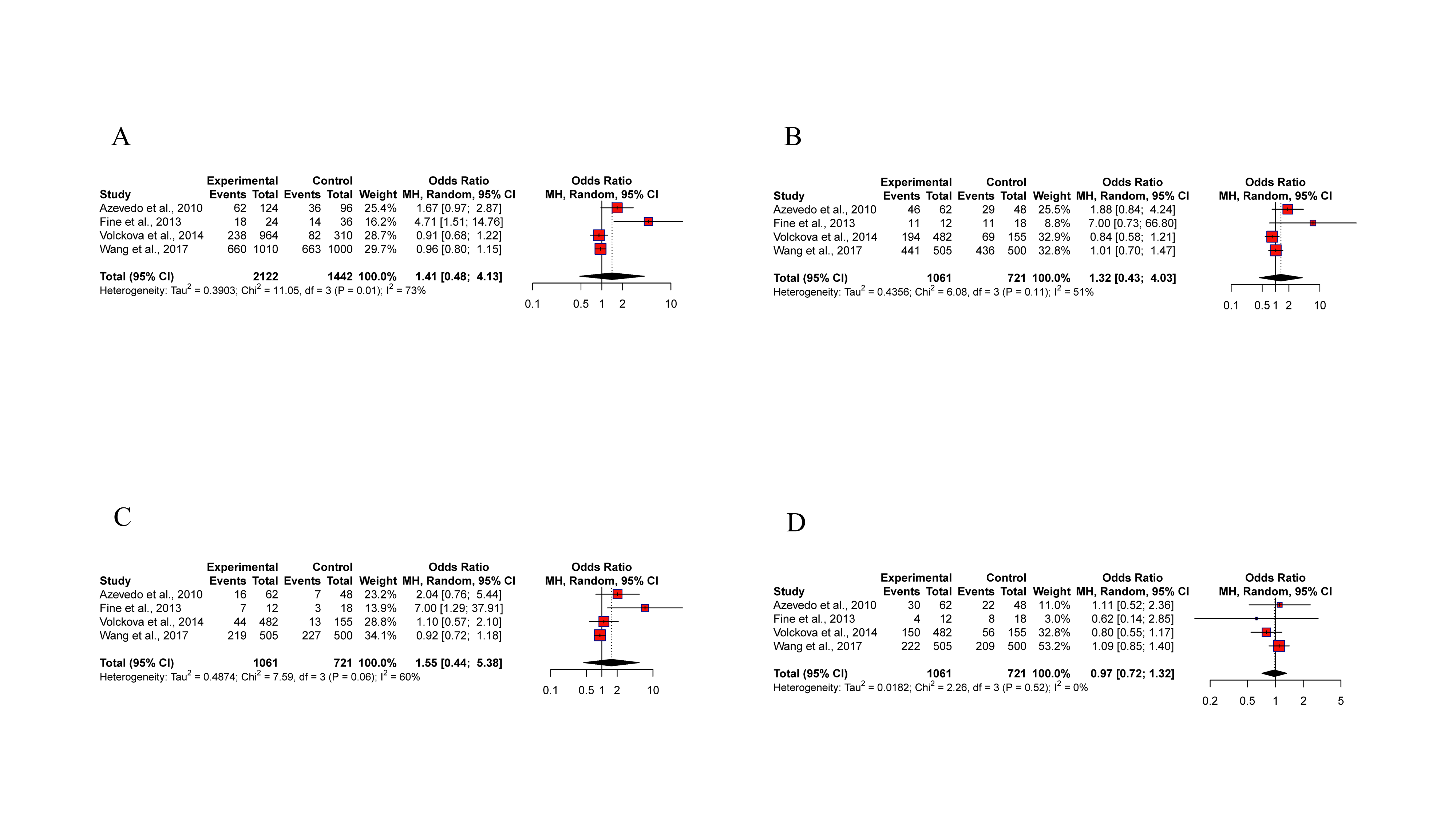

Supplement: Supplementary file 2 — Additional file 2: Figure S2. Forest plot for meta-analysis of the association of rs1126478 with dental caries using other genetic models. A) Allelic model; B) Dominant model; C) Recessive model and D) Co-dominant model. Each study is represented by a square whose area is proportional to the weight of the study. The overall effect from meta-analysis is represented by a diamond whose width represents the 95% CI for the estimated OR. OR, odds ratio; CI, confidence interval. [file 12881_2020_1029_MOESM2_ESM.tif]
